# Effect of Hemodialysis on Eye Coats, Axial Length, and Ocular Perfusion Pressure in Patients with Chronic Renal Failure

**DOI:** 10.1155/2018/3105138

**Published:** 2018-02-08

**Authors:** Ling Wang, Gang Yin, Zhiying Yu, Nan Chen, Dabo Wang

**Affiliations:** ^1^Department of Ophthalmology, Affiliated Hospital of Qingdao University, Qingdao 266003, China; ^2^Qingdao Central Hospital, Qingdao 266042, China

## Abstract

**Purpose:**

To investigate changes in eye coats, axial length, and ocular perfusion pressure (OPP) in hemodialysis (HD) patients with chronic renal failure (CRF).

**Methods:**

We included HD patients who were diagnosed with CRF in our hospital from January to December 2015. Fifty-two patients met the inclusion criteria; all right eyes were used for observation. Systolic and diastolic blood pressures were recorded to calculate OPP. Approximately 30 minutes before and after HD, we recorded multiple parameters, including lens thickness (LT), axial length of vitreous (VAL), axial length of eye (EAL), ciliary body thickness (CBT), choroidal thickness (CT), and retinal thickness (RT).

**Results:**

After HD, OPP significantly decreased (*P* < 0.01, *F* = 7.023) and CBT became significantly thinner (*P* < 0.01, *t* = 3.461). CT was significantly thinner and differed among measurement locations (*P* < 0.01, *t* = 6.240; *P* < 0.01, *t* = 6.169; *P* < 0.01, *t* = 3.405, respectively, fovea, nasal, and temporal side 1500 *μ*m beside the fovea). Further, RT thickened and differed among measurement locations (*P* < 0.05, *t* = −2.265; *P* < 0.05, *t* = −2.624; *P* < 0.05, *t* = −2.220, respectively, fovea, nasal, and temporal side 1500 *μ*m beside fovea), whereas LT, VAL, and EAL significantly increased after HD (*P* < 0.05, *t* = −2.076; *P* < 0.01, *t* = −3.817; *P* < 0.01, *t* = −4.010).

**Conclusions:**

HD impacts the thickness of the eye coats and VAL, particularly affecting CBT, CT, and RT. OPP transiently decreases during HD, which may contribute to an ischemic state.

## 1. Introduction

Chronic renal failure (CRF) is an irreversible deterioration of renal function, which is characterized by numerous disorders that involve many organs (e.g., heart, bone, blood vessels, and peripheral nerves). Hemodialysis (HD) is one of the main treatment methods in patients with CRF; its main objective is to correct the excessive accumulation and abnormal distribution of body fluid. However, sometime during or after HD, patients will occasionally develop a sharp increase in intraocular pressure (IOP), followed by transient blurred vision, eye pain, headache, and other symptoms [1, 2]. HD can also cause macular edema, ischemic optic neuropathy, retinal hemorrhage, retinal detachment, and other changes [3–6]. Recent research efforts have begun to focus on changes in the biological structure of the eye in HD-related pathologies, particularly in terms of the cornea, choroid, and retinal thickness [7, 8].

The aim of this study was to observe ocular parameters of the eyeball (e.g., thickness of each eye coat structure, depth of anterior chamber, thickness of lens, and axial length of vitreous) and ocular perfusion pressure (OPP) changes in hemodialysis patients before and after HD, as well as to explore the possible mechanisms of these changes. Further, we attempted to provide a fundamental basis for protecting visual function during HD through stabilization of the biological structures of the eye and improvement of ocular blood supply.

## 2. Materials and Methods

### 2.1. Participants

Fifty-two patients, who were diagnosed as CRF and underwent HD in our hospital, were enrolled in this group (25 men, 27 women). General characteristics of patients are summarized in Table 1. Only the right eyes of the patients were examined.

Inclusion criteria were as follows:

- Patients who exhibited good compliance and appropriate levels of physical activity and had signed informed consents.
- Patients whose visual acuity was ≥0.3 on the logMAR scale.
- Patients whose IOP was 10–21 mmHg.

Exclusion criteria were as follows:

- Patients with a history of glaucoma or ocular hypertension or eye surgery
- Patients suffering from severe fundus lesions.
- Patients exhibiting media opacity that affects optical clarity.
- Patients diagnosed with diabetes.

### 2.2. Hemodialysis Method

Only morning session HD patients were included. All patients underwent 4 h HD sessions, 3 days per week, at a blood flow rate of 250 mL/min. Patients were treated using high-performance dialyzers: 4008S-type Fresenius HD machine (Germany) and a Campbell 8L reusable dialyzer (Sweden); patient blood was dialyzed against bicarbonate dialysate (1.5 mmol/L calcium). All patients exhibited arteriovenous fistulae and used a polysulfone hollow-fiber dialyzer (Fx80, Germany). The total body weight was measured before and after HD.

### 2.3. Measurement of Plasma Osmotic Pressure

Blood samples were obtained within 60 seconds before the start and end of HD then used to calculate plasma osmotic pressure (plasma osmotic pressure = 1.86 × Na + glucose ÷ 18 + urea nitrogen ÷ 2 + 9).

### 2.4. Measuring the OPP

IOP was measured 30 min before the start of HD, 2 h after HD, and 30 min after ending HD, using a handheld rebound tonometer. Simultaneously, systolic blood pressure (SBP) and diastolic blood pressure (DBP) were recorded. OPP was acquired according to the formula: OPP(mean) = ((4/9) × DBP) + ((2/9) × SBP)–IOP.

All pre- and post-HD measurements were performed by the same examiner to minimize bias.

### 2.5. Central Corneal Thickness (CCT) Measurement

CCT was measured using a corneal endothelial cell counter (TOPCON SP-3000P, Japan), 30 min before the start and end of HD; it was measured three times to determine the mean value.

### 2.6. A-Ultrasound of the Eye

Patients were placed in the supine position. After administration of a topical anesthetic (0.25% oxybuprocaine eye drop), the patient was allowed to fix his or her finger to maintain vision straight ahead. Then, the central anterior chamber depth (ACD) and lens thickness (LT) were measured by A-ultrasonography, 30 min before the start and end of HD. Ten measurements were performed, and the average value was used.

### 2.7. Ultrasound Biomicroscopy (UBM) Measurements

Patients were placed in the supine position, with constant indoor brightness. A total of 30 min before the start and end of HD, iris thickness (IT) and ciliary body thickness (CBT) were measured by UBM at 12 o'clock, 6 o'clock, 3 o'clock, and 9 o'clock positions; the average values of the four quadrants were used.

**Figure 1:**
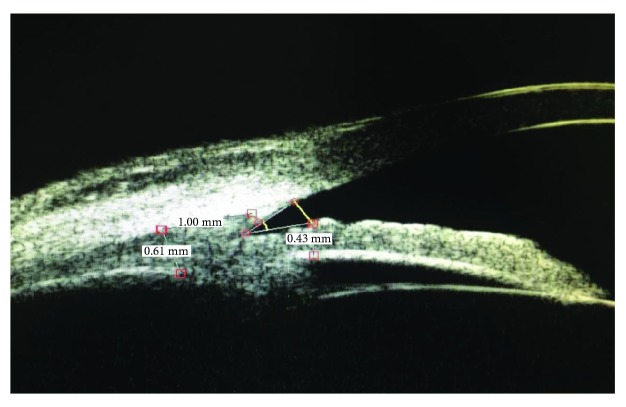
Iris thickness (IT) and ciliary body thickness (CBT) measurements by ultrasound biomicroscopy. IT was 0.43 mm and CBT was 0.61 mm.

- IT measurement: drawing a straight line perpendicular to the trabecular meshwork, 500 *μ*m in front of the scleral spur; intersecting with the anterior surface of the iris; and measuring the thickness of the iris at this point, as shown in Figure 1.
- CBT: measuring the thickness at the posterior 1 mm of the scleral spur, as shown in Figure 1.

### 2.8. Choroid and Retinal Thickness Measurements by Optical Coherence Tomography (OCT)

**Figure 2:**
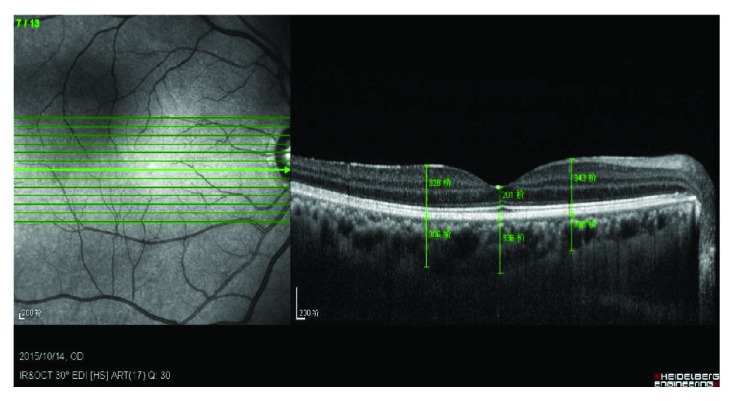
Choroid and retinal thickness measurements by optical coherence tomography.

A total of 30 min before the start and end of HD, choroidal thickness (CT) and retinal thickness (RT) were measured by OCT; these measurements were performed at the fovea, as well as at 1500 *μ*m distance from the fovea on both nasal and temporal sides. CT was measured from the outer portion of the high reflection line, corresponding to the retinal pigment epithelium, to the inner surface of the sclera. RT was measured from the retinal inner limiting membrane to the high reflection line of the retinal pigment epithelium, as shown in Figure 2. OCT scans were acquired before and after HD, without pupil dilation.

### 2.9. Statistical Analysis

All data were analyzed using the SPSS statistical package, version 19.0. The Kolmogorov-Smirnov test was used to assess normality of the data. SBP, DBP, IOP, and OPP were compared among groups, using the repeated measures one-way analysis of variance (ANOVA). Other variance comparisons were performed utilizing the paired-sample *t*-test. The Pearson coefficient test was used to determine correlations among changes in IOP and CCT, changes in eye coat thicknesses (i.e., cornea, iris, ciliary body, and choroid, retina), and the total amount of HD ultrafiltration. *P* values of <0.05 were considered to be statistically significant.

### 2.10. Ethical Approval

Ethical approval was obtained from the Medical Ethics Committee of the Affiliated Hospital of Qingdao University, before the start of the study. We certify that all applicable institutional and governmental regulations concerning the ethical use of human volunteers were followed during this research.

## 3. Results

### 3.1. Effect of Hemodialysis on Plasma Osmotic Pressure and Blood Biochemical Parameters (Table 2)

Plasma osmotic pressure after HD was significantly lower than plasma osmotic pressure before HD (*P* < 0.05, *t* = 3.041).

### 3.2. Effect of hemodialysis on SBP, DBP, IOP, and OPP (Table 3)

SBP in three groups was analyzed by one-way ANOVA (*F* = 6.175) and revealed significant differences (*P* < 0.05). SBP decreased significantly after 2 h of HD (*P* < 0.05) then returned to its pre-HD levels within 30 min after HD (*P* > 0.05).

DBP was analyzed by one-way ANOVA (*F* = 1.024); there were no statistical differences among the three groups (*P* > 0.05).

One-way ANOVA of IOP (*F* = 0.495) showed that there were no statistical differences among the three groups (*P* > 0.05).

One-way ANOVA of OPP (*F* = 7.023) showed significant differences among the three groups (*P* < 0.01). Compared with pre-HD levels, OPP significantly decreased 2 h after HD (*P* < 0.01); after the end of HD, OPP returns to its pre-HD levels, such that there was no significant difference between the two groups (*P* > 0.05).

### 3.3. Effect of Hemodialysis on the Structure of the Eye Coats (Table 4)

After the end of HD, CBT became significantly thinner (*P* < 0.01, *t* = 3.461). Compared with pre-HD measurements, CT was significantly thinner at all measurement points (*P* < 0.01, *t* = 6.240; *P* < 0.01, *t* = 6.169; *P* < 0.01, *t* = 3.405, respectively, fovea, nasal side 1500 *μ*m from the fovea, and temporal side 1500 *μ*m from the fovea). After HD, the RT was significantly thickened at all measurement points (*P* < 0.05, *t* = −2.265; *P* < 0.05, *t* = −2.624; *P* < 0.05, *t* = −2.220, respectively, fovea, nasal side 1500 *μ*m from the fovea, and temporal side 1500 *μ*m from the fovea). Although CCT and IT both decreased after HD, these changes were not statistically significant.

### 3.4. Effect of Hemodialysis on Ocular Axis-Related Parameters (Table 5)

LT significantly increased after HD (*P* < 0.05, *t* = −2.076). VAL and EAL both significantly increased after HD, relative to their pre-HD values (*P* < 0.01, *t* = −3.817; *P* < 0.01, *t* = −4.010). ACD slightly decreased after HD, but the difference was not statistically significant.

### 3.5. Analysis of Correlations

There were significant correlations between the total amount of ultrafiltration and differences in CBT (*r* = 0.640, *P* < 0.01), as well as between the total amount of ultrafiltration and differences in CT (*r* = 0.721, *P* < 0.01; *r* = 0.790, *P* < 0.001; *r* = 0.712, *P* < 0.01, respectively, fovea, nasal side 1500 *μ*m from the fovea, and temporal side 1500 *μ*m from the fovea). We did not observe a correlation between differences in IOP (before and after HD) and differences in CCT (*r* = −0.051, *P* > 0.05). No obvious correlations were observed between the total amount of ultrafiltration and differences in CCT (*r* = −0.107, *P* > 0.05), IT (*r* = 0.531, *P* > 0.05), or RT (*r* = −0.400, *P* > 0.05; *r* = 0.454, *P* > 0.05; *r* = −0.483, *P* > 0.05, respectively, fovea, nasal side 1500 *μ*m from the fovea, and temporal side 1500 *μ*m from the fovea).

## 4. Discussion

As an important treatment for patients with CRF, the side effects of hemodialysis have attracted much attention from health researchers. HD can cause a series of changes in the eye, including changes in refractive status, onset of dry eye, conjunctival calcification, banded keratopathy, lens opacity, and optic neuropathy [9, 10]. Moreover, there is a sharp increase in IOP during HD [11–14]. Recent studies have focused on post-HD changes in CCT, CT, RT, and OPP, in patients with CRF; notably, these studies have reported variable findings in some parameters [7, 8, 15], such that the effect of HD on these parameters is yet not clear. Here, we observed ocular parameters of the eyeball (i.e., thickness of each eye coat structure, ACD, LT, and vitreous axial length) and OPP changes in patients with CRF before and after HD in order to explore the possible mechanism of these changes.

During HD, toxic substances in the blood, such as urea nitrogen and creatinine, are dispersed into the hemodialysis solution, which causes a decrease in plasma osmotic pressure. Concurrently, ultrafiltration causes gradual reduction of extracellular fluid, thereby raising the osmotic pressure of the extracellular fluid. Subsequently, intracellular fluid outflow is altered to buffer extracellular osmotic pressure, resulting in a state of relative water shortage within tissues. Because of individual differences, the time for recovery of plasma osmotic pressure is different among patients; in some patients, plasma osmotic pressure returns to its pre-HD levels shortly after completion of HD. In this study, plasma osmotic pressure was measured only 60 sec after completion of HD. Although the plasma composition was not yet stable at this early stage, plasma osmotic pressure at this stage can reflect its variation during and after HD. Because of changes in plasma osmotic pressure, as well as the use of ultrafiltration in HD, the eye coat may be thinner. In previous studies, Dinc et al. [8] observed that CCT significantly decreased after HD. Yang [7] reported that the average CCT was significantly thinner and was associated with total weight loss after HD; however, changes in the thickness of the retinal nerve fiber layer were not obvious in that study. Jung et al. [10] also observed a significant thinning of the CCT after HD, using anterior OCT. In our study, CBT and CT both decreased after HD. Although post-HD CT and IT values were both less than pre-HD values, neither difference was statistically significant. We suspect that the avascularity of the cornea and the fewer vessels of the iris may contribute to this minimal thickness change. Because the retina is a nervous system with complex self-regulatory function, HD can cause retinal edema. Retinal thickening caused by edema was much greater than the thinning caused by dehydration; thus, we measured RT at each measurement point and found that all increased.

During HD, the urea nitrogen in the lens could not decrease at the same rate as the urea nitrogen in the blood, which resulted in an imbalance of osmotic pressure between the lens and aqueous humor; thus, the lens absorbed water and expanded, resulting in an increase in LT. Post-HD VAL was significantly greater than the pre-HD VAL. Concurrently, plasma osmotic pressure decreased, along with the plasma osmotic pressure of the eye coats; subsequently, water entered the vitreous cavity, leading to an increase in the length of the vitreous axis. The effect of HD on ACD is more complex: the increase of LT and forward positioning of the lens iris diaphragm may cause a shallow anterior chamber. However, the iris root and ciliary body were both thinner, which should cause a widening of the anterior chamber. Thus, there was no significant change in ACD after HD, in our study. Because of the changes in LT and VAL, axial length of the eye noticeably increased after HD.

Conflicting results have been reported concerning the effects of HD on IOP. In most studies, HD either causes increased IOP or causes no change in IOP [16–18]; a small number of studies have shown that HD can reduce IOP [19, 20]. A large number of cases reported sudden severe damage to visual function, including blindness, by dramatic intra- or post-HD increases in IOP [11–14]. The exact effects of HD on IOP are still unclear. In past studies, different methods for patient selection, patient grouping, or hemodialysis, as well as differences in hemodialysis equipment and time points analyzed, may influence the relationships between IOP and other factors, through variable assessment of the relationship between HD and IOP. In our current study, we found no significant difference in IOP at 2 h after HD and after the end of HD, compared with IOP before the start of HD. Further, we did not find a significant correlation between IOP and decreased CCT. We suspect that during HD, LT increased and the lens diaphragm moved forward, contributing to a shallow anterior chamber and elevation of the IOP. However, HD is a relative dehydration process, which causes tissues and cells to assume a relatively dry state. This may contribute to the thinning of the iris and ciliary body, thereby widening the anterior chamber angle and leading to a decreased outflow of aqueous humor; this would be expected to decrease IOP. This process may contribute to balancing of IOP during HD. The normal structure of the anterior chamber angle has a certain compensatory ability, and the formation and discharge of aqueous humor are in a balanced state. Thus, the IOP fluctuation caused by HD is not obvious.

HD is known to cause unfavorable side effects on systemic parameters, frequently including hypotension [21]. During a single session, removal of 2–4 L of fluid is probably the most important factor in dialysis-induced hypotension and the corresponding decrease in body weight. In this study, we found that SBP and body weight decreased significantly after HD, but that DBP did not significantly change. Autonomic nervous system dysfunction, which is common among dialysis patients, may contribute to this pathogenesis [22].

OPP represents the pressure of blood flowing in ocular blood vessels and is defined as the difference between arterial pressure and IOP, which is an important factor in regulating blood flow within the eye. OPP is calculated using blood pressure and IOP [23]. In this study, we used OPP as an indicator to evaluate the status of ocular blood supply. We found that the OPP of HD patients demonstrated a trend for reduction from the beginning to 2 h after HD but then returned to the pre-HD state. Thus, HD caused transient reduction in OPP, which may contribute to a state of ischemia. Studies have shown that if the capillary bed can be regulated, a change in OPP does not cause a change in blood flow [24–26]. If the capillary bed cannot self-regulate or exhibits weak adjustment ability, small changes in OPP will lead to changes in blood flow. However, vascular calcification and vasomotor dysfunction are present in most HD patients, as a result of systemic diseases and side effects of HD [27], thereby weakening the adjustment ability of the vascular bed. Particularly in HD patients with glaucoma or other ocular ischemic diseases, clinicians should carefully evaluate OPP changes to prevent visual function damage caused by HD.

In summary, the effect of HD on ocular parameters is mainly manifested in eye coat thinning, especially in the ciliary body and choroid. The retina is a nervous system with complex self-regulatory function; however, HD can cause retinal edema, and retinal thickening caused by edema was much greater than the thinning caused by dehydration. Because the choroid is rich in blood vessels, it is the main oxygen and nutrient supply for the outer retina. Decreased choroidal thickness from HD may affect retinal metabolism. There was no significant change in the IOP in our HD patients, but the OPP of these patients transiently decreased during HD, which may contribute to an ischemic state within the eye. Therefore, HD patients should aim to minimize fluctuations in blood pressure during HD. Clinicians should work to actively improve vascular conditions within HD patients, to slow the process of vascular sclerosis and calcification and to prevent vascular sclerosis or obstruction caused by weakened automatic regulation of ocular vessels, in order to protect visual function.

## 5. Conclusions

HD impacts the thickness of the eye coats and the vitreous axial length, including the thickness of the ciliary body, choroid, and retina, which may contribute to a decrease in ocular blood supply and retinal edema. OPP transiently decreases during HD, which contributes to an ischemic state within the eye. Thus, clinicians should work to minimize fluctuations in blood pressure during HD, in order to prevent this damage.

## Figures and Tables

**Table 1:** General characteristics of the patients.

Variable	Mean	SD	Range
Age (years)	52.4	10.1	40–68
Hemodialysis duration (year)	6.1	1.9	4–10
Total amount of ultrafiltration (mL)	2318	612.3	1500–3100
Weight loss (kg)	2.10	0.91	0.95–2.95

**Table 2:** Effect of hemodialysis on plasma osmotic pressure and blood biochemical parameters.

Variable	Urea nitrogen (mmol/l)	Glucose (mmol/l)	Na+ (mmol/l)	Creatinine (*μ*mol/l)	Plasma osmotic pressure (mOsm/kg H_2_O)
Pre-HD	26.06 ± 5.48	5.96 ± 1.19	138.18 ± 2.40	909.22 ± 119.93	279.37 ± 6.16
Post-HD	8.92 ± 3.79	7.85 ± 3.25	137.98 ± 4.35	395.52 ± 118.92	270.54 ± 7.32

**Table 3:** Effect of hemodialysis on systolic blood pressure, diastolic blood pressure, intraocular pressure, and ocular perfusion pressure.

Variable	Pre-HD	2 h of HD	Post-HD
SBP	135.19 ± 17.28	123.28 ± 18.56	133.03 ± 18.89
DBP	76.98 ± 14.02	74.26 ± 16.90	77.84 ± 15.07
IOP	16.87 ± 3.92	17.24 ± 3.64	17.42 ± 4.51
OPP	53.75 ± 7.80	50.67 ± 9.89	55.23 ± 9.73

**Table 4:** Effect of hemodialysis on the structure of the eye coats.

Variable	Pre-HD	Post-HD	*t*	*P*
CCT	0.506 ± 0.025	0.504 ± 0.023	1.252	>0.05
IT	0.380 ± 00.64	0.372 ± 0.061	1.607	>0.05
CBT	0.684 ± 0.059	0.668 ± 0.057	3.461	<0.01
CT (fovea)	267.97 ± 99.17	255.87 ± 95.59	6.240	<0.01
CT (nasal side 1500 *μ*m beside the fovea)	197.84 ± 95.43	186.29 ± 91.44	6.169	<0.01
CT (temporal side 1500 *μ*m beside the fovea)	233.37 ± 84.88	219.66 ± 83.04	3.405	<0.01
RT (fovea)	207.88 ± 23.47	209.94 ± 24.41	−2.265	<0.05
RT (nasal side 1500 *μ*m beside the fovea)	341.42 ± 23.32	343.88 ± 23.94	−2.624	<0.05
RT (temporal side 1500 *μ*m beside the fovea)	312.52 ± 22.90	314.15 ± 23.97	−2.220	<0.05

**Table 5:** Effect of hemodialysis on ocular axis-related parameters.

Variable	Pre-HD	Post-HD	*t*	*P*
ACD	2.74 ± 0.24	2.70 ± 0.29	1.439	>0.05
LT	4.85 ± 0.41	4.90 ± 0.43	−2.076	<0.05
VAL	15.43 ± 0.82	15.48 ± 0.82	−3.817	<0.01
EAL	23.04 ± 0.79	23.10 ± 0.79	−4.010	<0.01
